# Supplementary material for: Improving the acceptability of social robots: Make them look different from humans
Source: PLoS One. 2023 Nov 17;18(11):e0287507. doi: 10.1371/journal.pone.0287507 (PMC10656010; doi:10.1371/journal.pone.0287507)
Supplement: Attachment — Submitted filename: review.docx [file pone.0287507.s001.docx]

This is an interesting paper and certainly timely. My main problems/confusion are with the presented analysis of the data.

Page 11/17: Human resemblance. How exactly is the Human resemblance a 2x2 comparison? In fact, the authors perform two separate one-factorial ANOVAs. I have a couple of problems with understanding this analysis.

I guess the basic problem is that the authors label and treat factors based on the manipulations they did. that is “extremity” (upper, lower) x “type of manipulation” (4 types). This is not really good, because in fact these are only operationalization and not hypotheses-based factors. It also not correct, because it is not really crossed. What the author really want to do is to test hypotheses about simulatablity, efficiency and maybe consistency.

So actually it would be simulatability (high vs. low) vs efficiency (high vs low), although it seems that the authors have no hypotheses about efficiency in low simulatability. However, they offered two different types of low simulatable lower and upper extremities. I hope also with efficiency ideas in mind?! So it would be:

|  | Efficiency |  |
| --- | --- | --- |
| Simulatability | Low | High |
| Low | Floating / Attracting | Rolling / Hovering |
| High | Jumping / Pinching | Walking / Gripping |

In each cell is the further “technical factor” extremities (upper, lower), so it should be a 2x2x2 ANOVA. The hypotheses for resemblance: Main effect simulatability, maybe main effect efficiency, no main effect for extremity. An interaction effect simul x eff would be possible, if eff is only relevant for high simul, for example (as somewhat hinted at by the authors). To my mind, this is the analysis to be done, not two different one factorial ANOVAS. Another question is, whether the ANOVA is a repeated one? If I understood correctly, each participant produces ratings for each robot through comparison (hence for each factor level). Isn’t that dependent data then? You kind of switch to the robot as unit of analysis (a materials analysis) instead of participants ratings (subject analysis) (maybe because the authors are roboticists rather than social researcher, yet this is social research). In other words, the authors N is not the number of participants, but the number of robots. The variance from different ratings among the participants is lost by this, ins’t it. It is all about the differences among the robots.

A slight problem remaining with this is that it ignores the potential accentuating effects of combining upper and lower extremity. There are consistent robots (both extremities simulatable or not simulatable) and inconsistent robots (mixed extremities). It is not unlikely that this will have an effect and I would include according hypotheses explicitly. In the later analysis of all robot ratings they include this. However, the analysis is hard to follow. I am not sure, where the crucial comparison between consistent and inconsistent robot actually is; all seems reconstructed through post-hoc comparisons. This is also due to not actually introducing the factor “consistency” explicitly. I believe it should be treated as an factor all along. All in all, it makes it very complicated and I believe would have been better maybe not to combine upper and lower. So one possibility would also to only focus on one extremity and to hold the other constant for the analysis above. So for example only use robots that hover and put in the data for all variations of upper extremities in a 2x2 scheme above. However done, it must get more transparent and easier to understand.

Page 12/18. Social acceptability. This analysis should follow the factorial logic outlined above, or at least be always similar to the analysis strategy used in resemblance. In fact, the only difference is the measure (choice versus rating). Again the authors present an analysis with robots as unit of analysis. I find that strange. At the end of the day, each participant chose a certain social response to each level of factor, the robots are only operationalization. So I want to actually know the differences in relative and/or absolute frequencies for each response per factor level and not per robot (even if certain robots operationalize certain factor levels). In a way, the authors change their dependent variable (their measure, the different responses) to their independent variable and the robot ratings to their dependent variable. This is against the experimental logic. In fact, as showed above: the robots are operationalization of factor levels. The ratings of human resemblance are a measure, which should respond to the differences in factor levels (represented by robots). In the same logic, social acceptability should vary with factor levels. Instead the data is analyzed in a correlational manner now, although it originally had been an experiment.

The same happens in the final analysis.

To sum, the purpose of the paper is clear and the initial experimental design is in principle sound. However, the analysis does not follow the logic of the initial design. It mixes factorial and correlational analysis in way that makes it hard to follow. In addition, it remains unclear of whether all results remain stable, if the analysis stays in one logic, since this is very hard to tell from the way the data is presented. This is also due to the fact that descriptives are not reported in a way to understand them in detail (and to use them further in a meta-analysis, for example). The authors chose to present all data in figures, which is of course helpful, but make it impossible to get the true values (one has to guess at least a little). There are “error bars” in the figures, but again …

I believe that the analysis must be completely redone following the same factorial design (see above) for EACH measure and the matching ANOVAS (or frequency statistics, if proportions are problematic) accompanied by an interaction graph.
