# Supplementary material for: Improving the acceptability of social robots: Make them look different from humans
Source: PLoS One. 2023 Nov 17;18(11):e0287507. doi: 10.1371/journal.pone.0287507 (PMC10656010; doi:10.1371/journal.pone.0287507)
Supplement: Attachment — Submitted filename: Response to reviewers.docx [file pone.0287507.s002.docx]

Reviewer #1:

*Dear referee,*

*Thank you for your time and effort to understand and comment on our paper.*

*We followed your suggestions and the recommendation of the editor and entirely re-analyzed our data. We believe that the new analysis considerably improved the understanding of the result section. We hope that our revision will live up to your expectations.*

*As you correctly pointed out, simulatability and “efficiency” are two dimensions of our framework when we designed the robots. However, while simulatability is rather easy to operationalize based on the physical properties of the robots only (simulatable vs. non-simulatable), “efficiency” is not. This is particularly true for robots with non-simulatable functions, although by choosing functions such as “rolling; attracting” vs.“floating; hovering” we created a hierarchy based on familiarity with these functions (“rolling; attracting” more familiar than “floating; hovering”). However, as you further pointed out, the robots are just an operationalization of some arbitrary features that cannot be applied to other studies as they depend on our depiction of these features. We thought that the best attempt to improve on this point is to ask participants to rate robots on a psychological scale (i.e., human resemblance) and then build on the scales' theoretical implications. Therefore, we opt to use these ratings rather than the “efficiency” manipulation as independent variable; also because the ratings allow relating the robots on an interval scale (while “efficiency” is an ordinal scale). Unless there is a counter argument, we therefore would like to keep human resemblance as an independent variable to predict a) the perception of essentialized human qualities and b) the robots’ social acceptance.*

*In the revised version of our MS we separated the result section into three parts (note that for the different measures we tested* ***different groups of participants****):*

- *Part (1) serves to characterize the robots. In this part we report the results of the human resemblance ratings and show that these ratings clearly distinguish between robots with consistent simulatable and consistent non-simulatable functions. We then state that we will use this rating as independent variable to predict the perception of essentialized qualities and the social acceptance of the robots.*
- *In Part (2) we built a full model of the dependent variable* ***quality****, where simulatability and resemblance are the independent variables (qualities ∼ simulatability + resemblance + simulatability × resemblance); where × denotes the interaction between two independent variables.*
- *In Part (3), to test the effect of robot-characteristics on social responses (‘inclusion’, ‘exclusion/abuse’ and ‘ignore’) we used a generalized linear mixed model (GLMM) with a logit link as implemented in R's lme4::glmer (Bates et al., 2015). For the analyses of the group of 16 robots, a given response (e.g. ‘inclusion’), which was represented as a binary variable (chosen or not on each trial), was modeled as a logistic function of fixed effects, i.e. resemblance, simulatability, qualities, and the interaction between simulatability and resemblance. Resemblance and qualities are continuous variables, simulatability was divided into 2 binary categorical variables: the simulatability of upper and the simulatability of lower extremities (coded as: simulatable = 1 and non-simulatable = 0). For the analyses of the group of 8 robots and the groups of 4 robots with consistent SIM or non-SIM functions, the simulatability of upper and lower extremities were combined into one single binary variable. Random effects were selected to maximize the possible random effects structure.*

*Please let us know if you agree to these analyses.*

*Below we also report results conducted using the ANOVA design that you proposed. As you will see, the different analyses converge to the same results.*

*In the following we will reply to your comments one by one.*

*---------*

**This is an interesting paper and certainly timely. My main problems/confusion are with the presented analysis of the data.**

**Page 11/17: Human resemblance. How exactly is the Human resemblance a 2x2 comparison?**

*N, L, & L: Human resemblance ratings are obtained through the 2x2 comparisons of each robot with each of the other 15 robots. Participants are requested to specify which of two robots resembles more a human. The score for a given robot thus varies between 0 (never chosen) and 15 (always chosen). This procedure is specified in the method section.*

*In the revised version we deleted the term “2x2 comparisons” in the sub-titles of the analyses sections.*

---------

**In fact, the authors perform two separate one-factorial ANOVAs. I have a couple of problems with understanding this analysis.**

**I guess the basic problem is that the authors label and treat factors based on the manipulations they did. that is “extremity” (upper, lower) x “type of manipulation” (4 types). This is not really good, because in fact these are only operationalization and not hypotheses-based factors. It also not correct, because it is not really crossed. What the author really want to do is to test hypotheses about simulatablity, efficiency and maybe consistency.**

**So actually it would be simulatability (high vs. low) vs efficiency (high vs low), although it seems that the authors have no hypotheses about efficiency in low simulatability. However, they offered two different types of low simulatable lower and upper extremities. I hope also with efficiency ideas in mind?! So it would be:**

|  | **Efficiency** |  |
| --- | --- | --- |
| **Simulatability** | **Low** | **High** |
| **Low** | **Floating / Attracting** | **Rolling / Hovering** |
| **High** | **Jumping / Pinching** | **Walking / Gripping** |

**In each cell is the further “technical factor” extremities (upper, lower), so it should be a 2x2x2 ANOVA.**

*N, L, & L: We conducted the proposed analysis (including all 16 robots).*

*NOTE THAT THIS IS NOT THE ANALYSES THAT WE RETAINED FOR OUR MS*

*In the regression analysis, we classified simulatability and efficiency into two levels respectively (high and low), and used these factors to predict ratings of human resemblance and essentialized quality (Table 1). The results show that the predictors predict the two DVs for both upper- and lower-extremities, except for the main effect of efficiency on resemblance for the upper extremities.*

***Table 1.*** *Main effects and interaction of simulatability and efficiency on essentialized human quality and human resemblance. The first column to the right of df is the result of the regression analysis with upper extremities, and the second column is the result of the regression analysis with lower extremities. (×) denotes the interaction.* * *p* < .05, ****** *p* **<** .01, ******* *p* < .001

*In a second analysis we examined the effects of simulatability and efficiency on social responses, with a design matrix similar to that of the above analysis, but in a GLMM with a logit link (similar to the one used in the revised version of our manuscript). Table 2 gives the results of these analyses separately for “include”, “exclude/attack”, and “ignore” responses. For “include” and “exclude/attack” responses the interaction between simulatability and efficiency is significant. This supports the notion that the two types of responses vary with efficiency in an opposing way for robots with simulatable and non-simulatable functions. For “ignore” responses there is no interaction but a significant main effect of simulatability for lower extremities, supporting the notion that higher “ignore” responses are given to robots with non-SIM functions.*

***Table 2.*** *Main effects and interaction of simulatability and efficiency on social behavior. The first column to the right of effects is the result of the regression analysis with upper extremities, and the second column is the result of the regression analysis with lower extremities. (×) denotes the interaction. † p < .1,* * *p* < .05, ****** *p* **<** .01, ******* *p* < .001

**A slight problem remaining with this is that it ignores the potential accentuating effects of combining upper and lower extremity. There are consistent robots (both extremities simulatable or not simulatable) and inconsistent robots (mixed extremities). It is not unlikely that this will have an effect and I would include according hypotheses explicitly.**

*N, L, & L: Thank you for this suggestion. In the revised version of the MS we follow your suggestion and take into account “simulatability” as a factor by considering three ordinal levels (robots with consistent simulatable function (SIM), robots with consistent non-simulatable functions (non-SIM)), and robots with hybrid functions (SIM paired with non-SIM). In this way, the effect of combining upper and lower extremities is taken into account.*

**---------**

**All in all, it makes it very complicated and I believe would have been better maybe not to combine upper and lower. So one possibility would also to only focus on one extremity and to hold the other constant for the analysis above. So for example only use robots that hover and put in the data for all variations of upper extremities in a 2x2 scheme above. However done, it must get more transparent and easier to understand.**

*N, L, & L: The analysis presenting the results holding one extremity and manipulating the other one is now shown in the manuscript (see Table 2a in MS). It is one part in a series of analyses on different responses (ignore vs non-ignore & inclusion vs non-inclusion) and robot groups (all, consistent, SIM and non-SIM robots). And based on that we focused on comparing robots with consistent SIM and non-SIM functions in particular, as interpreting the results for hybrid robots is not straightforward. In this way, the influence from upper, lower and from the combined functions of simulatability are more transparent. We'd be happy to hear further advice about the current solution.*

**---------**

**Page 12/18. Social acceptability. This analysis should follow the factorial logic outlined above, or at least be always similar to the analysis strategy used in resemblance. In fact, the only difference is the measure (choice versus rating). Again the authors present an analysis with robots as unit of analysis. I find that strange. At the end of the day, each participant chose a certain social response to each level of factor, the robots are only operationalization.**

*N, L, & L: please see the above Table 2.*

**---------**

**In fact, as showed above: the robots are operationalization of factor levels. The ratings of human resemblance are a measure, which should respond to the differences in factor levels (represented by robots). In the same logic, social acceptability should vary with factor levels. Instead the data is analyzed in a correlational manner now, although it originally had been an experiment.**

**The same happens in the final analysis.**

*N, L, & L: We no longer present this figure.*

**To sum, the purpose of the paper is clear and the initial experimental design is in principle sound. However, the analysis does not follow the logic of the initial design. It mixes factorial and correlational analysis in way that makes it hard to follow. In addition, it remains unclear of whether all results remain stable, if the analysis stays in one logic, since this is very hard to tell from the way the data is presented.**

*N, L, & L: We hope that the new analyses are better accessible.*

**This is also due to the fact that descriptives are not reported in a way to understand them in detail (and to use them further in a meta-analysis, for example). The authors chose to present all data in figures, which is of course helpful, but make it impossible to get the true values (one has to guess at least a little). There are “error bars” in the figures, but again …**

*N, L, & L: We now provide tables with the data next to the figures.*

**---------**

**I believe that the analysis must be completely redone following the same factorial design (see above) for EACH measure and the matching ANOVAS (or frequency statistics, if proportions are problematic) accompanied by an interaction graph.**

*N, L, & L: We combined this request with the recommendation of the editor and used a GLMM to analyze the social responses. Please let us know your opinion about the new analyses.*

—------------------

Reviewer #2:

*Dear referee,*

*Thank you for your time and effort to understand and comment on our paper.*

Unfortunately, information about the number of participants, participants’ age, and socio-economic background is not provided in the paper. Maybe, the acceptance of the social robots will vary depending on the education level of the participants, too. There is no information in the paper in this respect. Also, the authors do not provide information as to how the participants have been chosen in order to be included in the experimental groups.

*N, L, & L: Thank you for this remark. By mistake, the section with the description of our participants was entirely missing in the first version. Information about age and gender is now provided. The exclusion criterion was that participants should not have neurological or psychiatric disorders. This is now specified.*

It would be a good idea if the authors elaborate more on the interpretation of the results from the statistical analysis.

*N, L, & L: In the new version, in line with requirements from referee 1 and from the editor, we reanalyzed all our data. We also elaborate the interpretation of the results.*

As a minor technical remark could be mentioned that the text in some figures is difficult to read.

*N, L, & L: Thank you for this remark. We modified the figures to improve readability.*

The study proposed in the paper is interesting and the paper is well organized. Although the experiments are conducted with computer animated robots, this study could be useful for the developers of novel social robots, because it shed light on the relationship between the robot design and the level of the acceptance by the humans.

*N, L, & L: Thank you very much for this remark. Actually, in a recent meta-analysis by Roesler et al. (2021) on the effect of anthropomorphism in human-robot interaction, no effect of the physical presence of the robot (as opposed to a depiction) was found. In our revision, we now refer to this meta-analysis.*
